# Study on the Potential Molecular Mechanism of Keloid Disease Associated With Single Cell Combined Mendelian Randomization

**DOI:** 10.1111/jocd.70849

**Published:** 2026-04-15

**Authors:** Hui‐Hui Wu, Yong‐Xia Meng, Juan‐Hua Liu, Di‐Qing Luo, Mu‐Kai Chen, Yu‐Kun Zhao

**Affiliations:** ^1^ Department of Dermatology, the First Affiliated Hospital Sun Yat‐Sen University Guangzhou China

**Keywords:** keloids, Mendelian randomization analyses, protein quantitative trait loci, single‐cell transcriptomics, *SRA1*, *SSR1*

## Abstract

**Background:**

Keloids are pathological scars with incompletely understood pathogenesis. This study aims to identify the key genes and regulatory networks potentially involved in keloid formation by integrating single‐cell transcriptomics (scRNA‐seq), protein quantitative trait loci (pQTL), Mendelian randomization (MR) analyses, colocalization, and comprehensive functional characterization.

**Methods:**

Single‐cell RNA sequencing data (GSE181297, GSE163973) and bulk transcriptomic data (GSE145725) were obtained from the GEO database. These datasets, which included both keloid lesions and normal scar samples from distinct individuals, were subjected to rigorous quality control and cell annotation. Intercellular communication was analyzed using CellChat, and co‐expression networks were constructed via hdWGCNA. To identify potential causal genes, MR analysis was performed by integrating pQTL data from the deCODE database with GWAS summary statistics (GCST90018874), followed by colocalization, sensitivity analysis, and reverse validation. Further functional characterization of potential key genes was conducted through Gene Set Enrichment Analysis (GSEA), immune infiltration analysis, transcription factor (TF) regulatory network inference, and pseudotime trajectory analysis.

**Results:**

92 659 high‐quality cells were retained, revealing seven major cell types with fibroblasts showing the most extensive intercellular interactions. Mendelian randomization identified 11 genes causally linked to keloid risk, with genetically determined downregulation of *SSR1* and *SRA1* associated with increased susceptibility. *SRA1* was enriched in cell cycle, nucleocytoplasmic transport, and ribosome biogenesis in eukaryotes, while *SSR1* was implicated in complement/coagulation cascades, the NOD‐like receptor signaling pathway, and viral protein interaction with cytokine and cytokine receptors. Immunoinfiltration, TF network and motif analysis, and pseudotime analysis further characterized their regulatory roles in immune microenvironment modulation and cell differentiation.

**Conclusions:**

This multi‐omics study identifies *SRA1* and *SSR1* as potential key protective genes in keloid pathogenesis. These genes may contribute to inhibiting disease progression through modulation of cell proliferation, the immune microenvironment, and cellular signaling pathways. These findings provide novel mechanistic insights and potential immunotherapeutic targets for keloids.

## 1 Introduction

Keloids are pathological scars that most commonly occur in individuals of color, with a reported prevalence ranging from 4.5% to 16% [1, 2]. The condition typically affects individuals under 30 years of age and shows no sex predilection [1, 2]. Clinically, keloids present as irregular, infiltrated plaques and nodules with a rubbery texture, often resembling crab claws [3]. These lesions frequently invade adjacent healthy skin beyond the confines of the original wound and do not undergo spontaneous regression [3]. In addition to cosmetic disfigurement, keloid disease can lead to decreased quality of life due to associated symptoms and complications, including pruritus, pain, hyperaesthesia, infection, ulceration, sinus tract formation, malignant transformation, and functional impairment [4, 5, 6, 7].

Keloid pathogenesis is complex, involving abnormal fibroblast proliferation, immune microenvironment dysregulation, and perturbations in gene regulatory networks [3, 8]. Previous genetic studies have implicated multiple susceptibility loci and genes including NEDD4, COL5A1, COL5A2, SERPINH1, HLA‐DRB1*15, HLA‐DQA1, HLA‐DQB1, among others [9, 10, 11, 12]. The microenvironment imbalance in keloid lesions primarily involves several key factors. These include the overexpression of cytokines such as TGF‐β, PDGF, TNF‐α, FGF, EGF, and VEGF; the aberrant activation of signaling pathways including TGF‐β/Smad, Wnt/β‐catenin, PI3K/AKT/mTOR, JAK/STAT, IL‐17, and Notch‐1/JAG‐1; and the increased infiltration of immune cells, particularly M2 macrophages, Treg cells, CD8+ T cells, Th2 cells, mast cells, NK cells, and dendritic cells [8, 13, 14, 15]. However, the exact mechanism of keloid formation remains unclear.

The treatment of keloids remains a significant clinical challenge, largely due to high recurrence rates and unsatisfactory therapeutic outcomes [1, 16, 17, 18]. A deeper understanding of the genetic basis and immune regulatory networks underlying keloid pathogenesis is therefore essential for the development of more effective management strategies.

Advances in computational genomics have enabled efficient microarray‐based discovery of critical genes and exploration of their functional roles [19]. Among these techniques, single‐cell RNA sequencing (scRNA‐seq) has emerged as a powerful tool capable of resolving transcriptomic heterogeneity at single‐cell resolution and is now widely applied in human disease research [20]. Mendelian randomization (MR) provides a framework for causal inference in genetic epidemiology by utilizing heritable genetic variants as instrumental variables (IVs) to assess causal relationships between modifiable risk factors and clinical outcomes [21]. When integrated with single‐cell transcriptomics and quantitative trait locus (QTL) data, such as protein QTLs (pQTL) or expression QTLs (eQTL), this approach enables the inference of direct causal pathways from gene products to disease pathogenesis, thereby advancing mechanistic discovery and the identification of potential therapeutic targets [22, 23].

In the present study, we therefore employed a multi‐omics strategy integrating single‐cell transcriptomics, pQTL data, MR, colocalization, and comprehensive functional characterization to identify key genes involved in keloid pathogenesis and to explore their roles in regulating cell proliferation, immune microenvironment dynamics, and transcriptional networks. Our findings provide novel mechanistic insights and potential therapeutic targets for keloid disease.

## 2 Materials and Methods

### 2.1 Data Acquisition

(1) Single‐cell RNA sequencing (scRNA‐seq) datasets GSE181297 and GSE163973 were obtained from the Gene Expression Omnibus (GEO) database (National Center for Biotechnology Information, NCBI) [24]. These datasets comprised nine samples with complete single‐cell transcriptional profiles, including four normal scar samples as controls and five keloid samples (Table S1) [25, 26]. Additionally, bulk RNA‐sequencing data (GSE145725, annotation platform GPL16043) were retrieved, encompassing 19 samples in total (10 controls, 9 disease cases).

(2) Exposure data: protein quantitative trait locus (pQTL) data were obtained from the deCODE database (2021 release; PMID: 34857953). This dataset contains genome‐wide association study (GWAS) summary statistics derived from 35 559 Icelandic individuals. In that study, plasma protein levels were quantified using 4907 aptamers.

(3) Outcome data: Summary‐level genome‐wide association study (GWAS) statistics for keloid were obtained from the GWAS Catalog (accession number: GCST90018874) [27]. This dataset, derived from participants mainly of European ancestry, included 668 cases and 481 244 controls. The summary statistics have been mapped to the latest human genome assembly and dbSNP build.

### 2.2 Single Cell Data Quality Control

The gene expression matrix was initially processed using the Seurat package [28]. Cell filtration was performed based on multiple quality metrics, including total unique molecular identifier (UMI) counts, number of detected genes, and the proportions of mitochondrial and ribosomal gene expression.

Specifically, the mitochondrial and ribosomal gene proportions were calculated as the fraction of expression derived from these gene sets relative to the total gene expression per cell. Cells exhibiting elevated mitochondrial or ribosomal gene proportions typically indicate low RNA content and may represent apoptotic or low‐quality cells. To exclude such cells, we applied the median absolute deviation (MAD) method. Variables exceeding 3 MADs from the median were flagged as outliers and removed. In addition, DoubletFinder (version 2.0.4) was employed to identify and exclude potential doublets within each individual sample, thereby enhancing the reliability of the downstream analyses [29].

### 2.3 Dimension Reduction, Clustering and Cell Annotation of Single‐Cell Data

Normalization was performed using the LogNormalize method implemented in the Seurat package, in which total expression per cell was scaled to 10 000 counts and subsequently log‐transformed. Cell cycle effects were quantified using the CellCycleScoring function. Highly variable genes were identified with the FindVariableFeatures function. To mitigate unwanted variation arising from mitochondrial and ribosomal gene content as well as cell cycle heterogeneity, the ScaleData function was applied for data standardization.

Principal component analysis (PCA) was then conducted for linear dimensionality reduction, and the relevant principal components were selected for downstream analyses. Batch effects were corrected using the Harmony algorithm, followed by nonlinear dimensionality reduction via the RunUMAP function, which implements the Uniform Manifold Approximation and Projection (UMAP) algorithm. For cell type identification, we referenced the CellMarker and PanglaoDB databases in conjunction with relevant literature. This was supplemented by automated annotation using SingleR, enabling accurate assignment of cell types and characterization of their respective marker genes.

### 2.4 Ligand Receptor Interaction Analysis (CellChat)

To investigate intercellular communication networks, we applied CellChat, a computational tool that enables quantitative inference and analysis of cell–cell signaling from single‐cell transcriptomic data [30]. CellChat integrates network modeling with pattern recognition techniques to identify and quantify communication probabilities between cell populations. It predicts key signaling sources and targets, as well as how signals and cells coordinate to regulate biological processes.

In this analysis, normalized single‐cell gene expression data were used as input, with previously defined cell subtypes incorporated as cell identity information. Intercellular communication was assessed by examining both interaction strength (weights) and the number of interactions (counts) between cell types. This approach enabled us to evaluate signaling activity and the relative influence of each cell population within the disease context.

### 2.5 High‐Dimensional Weighted Gene Co‐Expression Network Analysis (hdWGCNA)

The hdWGCNA provides built‐in capabilities for network inference, gene module identification, functional enrichment analysis, statistical testing of network reproducibility, and data visualization [31]. It offers a comprehensive framework for constructing gene co‐expression networks, identifying gene modules, performing functional enrichment, assessing network robustness, and generating visual outputs. Beyond conventional single‐cell RNA sequencing analysis, it also supports homotypic network analysis on long‐read single‐cell datasets. To initiate network construction, we applied the SetupForWGCNA function, which selected genes expressed in at least 5% of cells within the Seurat object. A soft‐thresholding power of 8 was chosen to establish a scale‐free topology. The resulting hierarchical clustering tree, visualized using PlotDendrogram, delineated the gene modules, with each leaf corresponding to a gene and color‐coded branches representing distinct co‐expression clusters. Finally, module eigengene (ME) profiles were extracted using the GetMEs function to display the expression patterns of each module.

### 2.6 MR Analysis

Single‐nucleotide polymorphisms (SNPs) associated with each gene at a genome‐wide significance threshold (*p* < 1 × 10^−5^) were selected as potential instrumental variables (IVs). To account for linkage disequilibrium (LD), we performed clumping and retained only independent SNPs (LD *r* ^2^ < 0.001) within a 10 000 kb window. Subsequently, the instrumental strength of all candidate IVs was systematically evaluated using the F‐statistic; only SNPs with an F‐statistic > 10 (the conventional threshold for avoiding weak instrument bias) were retained as valid IVs, and no SNPs with an F‐statistic < 10 were included in the final analysis. For causal effect estimation, four complementary MR approaches were employed to enhance the reliability of causal inferences: the inverse variance weighted (IVW) meta‐analysis (primary analysis, aggregating SNP‐level Wald ratios), MR‐Egger regression (accounting for directional pleiotropy under the Instrument Strength Independent of Direct Effect [InSIDE] assumption), the weighted median method (yielding consistent estimates even if up to 50% of IVs are invalid), and the weighted mode approach (with lower bias and reduced Type I error rates relative to MR‐Egger regression in specific scenarios). When only a single valid SNP was available as an IV, the Wald ratio method was applied for causal inference. These complementary methods jointly provided an overall estimate of the causal effect of cis‐ and selected trans‐gene expression in whole blood on disease risk. A leave‐one‐out sensitivity analysis was subsequently conducted to assess the robustness of the causal inference and to identify potentially influential SNPs.

### 2.7 Sensitivity Analysis

To evaluate the influence of individual genetic variants on the causal estimates, we conducted a leave‐one‐out sensitivity analysis within the MR framework. This approach iteratively excludes one SNP at a time and recalculates the pooled causal effect using the remaining variants, thereby identifying SNPs that disproportionately influence the overall result. For each iteration, a new point estimate and its corresponding 95% confidence interval were generated, enabling assessment of each SNP's impact on the consistency of the findings. The recalculated estimates from each leave‐one‐out iteration were then compared to the overall estimate derived from all SNPs. This comparison serves to determine whether any single SNP substantially alters the overall causal inference, thereby providing a measure of the robustness and stability of the results.

### 2.8 Colocalization

To perform colocalization analysis, we applied the coloc approach, integrating summary‐level quantitative trait locus (eQTL) data with genome‐wide association study (GWAS) summary statistics for keloid. A genomic window spanning 100 kilobases around the lead SNP was selected for calculating posterior probabilities.

In the coloc output, hypothesis H3 denotes that both traits—gene expression and keloid—are associated but driven by distinct causal variants, whereas H4 indicates a shared causal variant underlying both traits. A threshold of SNP.PP.H4 > 0.80 was applied to define significant co‐localization, suggesting a high likelihood of shared genetic causality.

### 2.9 Gene Set Enrichment Analysis

Based on the expression profiles of selected key genes, samples were stratified into high‐ and low‐expression groups. To investigate functional differences between these groups, gene set enrichment analysis (GSEA) was conducted [32]. Reference gene sets were obtained from version 7.0 of the Molecular Signatures Database (MSigDB), with a focus on annotated subtype‐specific pathways. Pathway‐level enrichment analysis was performed, and gene sets with an adjusted *P* value below 0.05 were considered statistically significant. Enriched pathways were subsequently ranked according to their normalized enrichment scores (NES) to evaluate the consistency and directionality of biological relevance. GSEA provides a robust framework for linking gene expression variation to disease‐related biological functions.

### 2.10 Immunoinfiltration Analysis

CIBERSORT is a widely used computational approach for characterizing immune cell composition within the tissue microenvironment [33]. Utilizing support vector regression, this method performs deconvolution on gene expression matrices to estimate the relative proportions of immune cell subtypes. The algorithm leverages 547 signature genes to distinguish 22 distinct human immune cell types, including subsets of T cells, B cells, plasma cells, and various myeloid lineages. In this study, we applied CIBERSORT to patient transcriptomic data to infer the relative abundance of these 22 immune cell populations. Subsequently, correlation analyses were performed to explore associations between the expression levels of key genes and the estimated immune cell fractions.

### 2.11 Transcription Factor Regulatory Network

Transcription factor prediction was performed using the R package RcisTarget, which evaluates regulatory potential based on DNA motif analysis [34]. For each motif, a normalized enrichment score (NES) is calculated relative to the total number of motifs present in the reference database. In addition to source‐annotated motifs, further annotations were inferred by assessing motif similarity and corresponding gene sequences. The initial step involves computing the area under the curve (AUC) for each motif–gene set pair, which reflects the motif's enrichment within a ranked recovery curve derived from the target gene set. NES values were subsequently derived from the distribution of AUCs, providing a quantitative measure of each motif's regulatory activity across the gene set.

### 2.12 Pseudotime Analysis

Single‐cell transcriptomic profiling has enabled the dissection of transcriptional regulation across complex physiological processes and highly heterogeneous cell populations. Such analyses facilitate the identification of genes that define specific cell types, mark transitional states during dynamic biological processes, or mediate phenotypic shifts between distinct cellular fates. In many single‐cell transcriptomic studies, gene expression is captured at asynchronous time points, with each cell representing a unique snapshot of the overall transcriptional progression.

Monocle addresses the challenge of reconstructing continuous biological processes from static snapshots by introducing pseudotemporal analysis [35]. This approach orders cells along a trajectory based on similarities in their gene expression states, thereby modeling dynamic processes (e.g., cell differentiation) without the need for synchronized sampling or time‐series experiments.

### 2.13 Statistical Analysis

All statistical analyses were conducted using R language (version 4.3.0), with *p* < 0.05 considered statistically significant.

## 3 Results

### 3.1 Single Cell Data Quality Control

To ensure consistent data quality across samples, we excluded cells with fewer than 500 detected genes. The filtering strategy was defined as follows: nFeature_RNA > 500; percent.mt ≤ median + 3 MAD; nFeature_RNA ≤ median + 3 MAD; nCount_RNA ≤ median + 3 MAD; percent.ribo ≤ median + 3 MAD. Here, nFeature_RNA denotes the number of genes detected per cell, nCount_RNA represents the total unique molecular identifier (UMI) counts, percent.mt indicates the proportion of mitochondrial gene expression, and percent.ribo reflects the proportion of ribosomal gene expression. Potential doublets were identified and excluded using the DoubletFinder package. Following all quality control steps, a final dataset comprising 92 659 high‐quality cells was retained for downstream analyses. Filtering results are illustrated in violin and scatter plots (Figure S1A,B). Subsequently, 2000 highly variable genes were selected for further analysis (Figure S1C). Data preprocessing steps including normalization, scaling, principal component analysis (PCA), and batch effect correction using Harmony integration were then applied (Figure S1D–F).

### 3.2 Cell Annotation and Cell Communication Analysis

**FIGURE 1:**
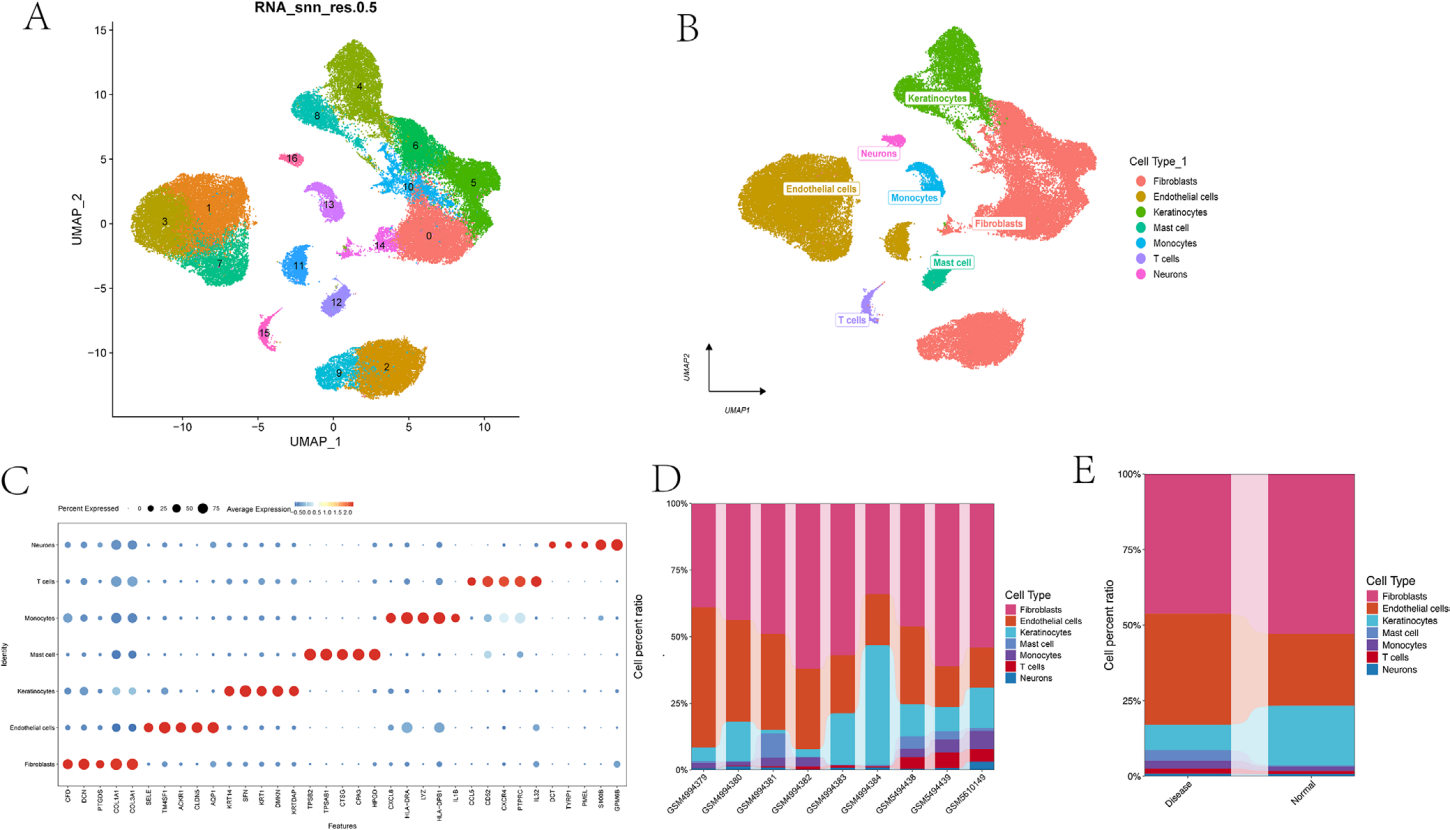
Cell type identification and annotation in normal scar control and keloid samples. (A) Uniform Manifold Approximation and Projection (UMAP) plot showing 17 distinct cell clusters identified following principal component analysis (PCA). (B) Annotation of the 17 clusters, which were classified into seven major cell types: Fibroblasts, endothelial cells, keratinocytes, mast cells, monocytes, T cells, and neurons. (C) Bubble plot depicting the expression levels of canonical marker genes across the seven annotated cell types. (D) Bar plot showing the distribution of the seven cell types across individual samples. (E) Comparison of cell type proportions between keloid samples and normal scar controls.

**FIGURE 2:**
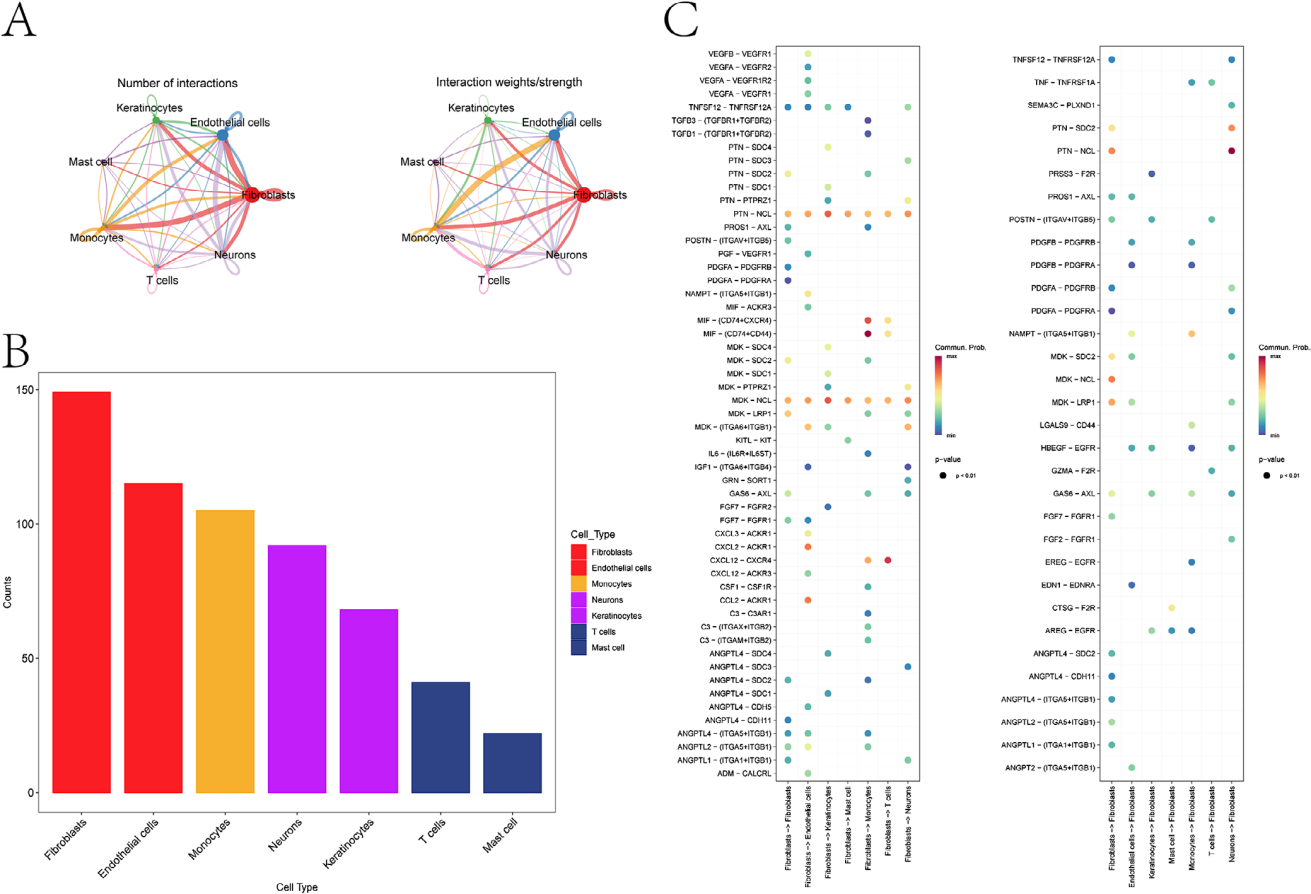
Cell communication analysis in keloid tissues. (A) Network visualization of intercellular interactions among the seven annotated cell types. Edge width represents the communication probability and signaling intensity between cell populations. (B) Quantification of the total number of interactions for each cell type within the communication network. Cell types are arranged in descending order from left to right, with fibroblasts exhibiting the highest interaction frequency. (C) Bubble plot displaying significant ligand–receptor pairs underlying the intercellular communication among the seven cell types.

Uniform Manifold Approximation and Projection (UMAP) dimensionality reduction revealed 17 distinct cell clusters (Figure 1A). These clusters were subsequently annotated into seven major cell types: fibroblasts, endothelial cells, keratinocytes, mast cells, monocytes, T cells, and neurons (Figure 1B). The expression patterns of canonical marker genes for each cell type are visualized in a bubble plot (Figure 1C). The distribution of these cell types across the nine individual samples, as well as a comparison between keloid disease and normal scar control groups, is shown in bar plots, respectively (Figure 1D,E). To investigate intercellular communication, we applied the CellChat package to analyze ligand–receptor interactions among the identified cell types. This analysis revealed that fibroblasts engage in extensive and robust interactions with a broad range of other cell populations, particularly endothelial cells, myeloid immune cells, and T cells (Figure 2A). Quantitative analysis of intercellular communication revealed that fibroblasts were the most frequent interactors, exhibiting a level of connectivity far exceeding that of other cell types (Figure 2B). Significant ligand–receptor pairs involving fibroblasts (*p* < 0.01) were identified, with the MIF‐(CD74 + CD44) and PTN‐NCL axes being the most prominent pathways (Figure 2C). Following reclustering of fibroblasts using PCA, Harmony, ElbowPlot, and FindClusters, we identified 12 distinct fibroblast subtypes (Figure S2A–D). Notably, subtype 3 was enriched in disease samples and was therefore designated as a disease‐specific fibroblast population (Figure S2E).

### 3.3 High‐Dimensional Weighted Gene Co‐Expression Network Analysis (hdWGCNA)

**FIGURE 3:**
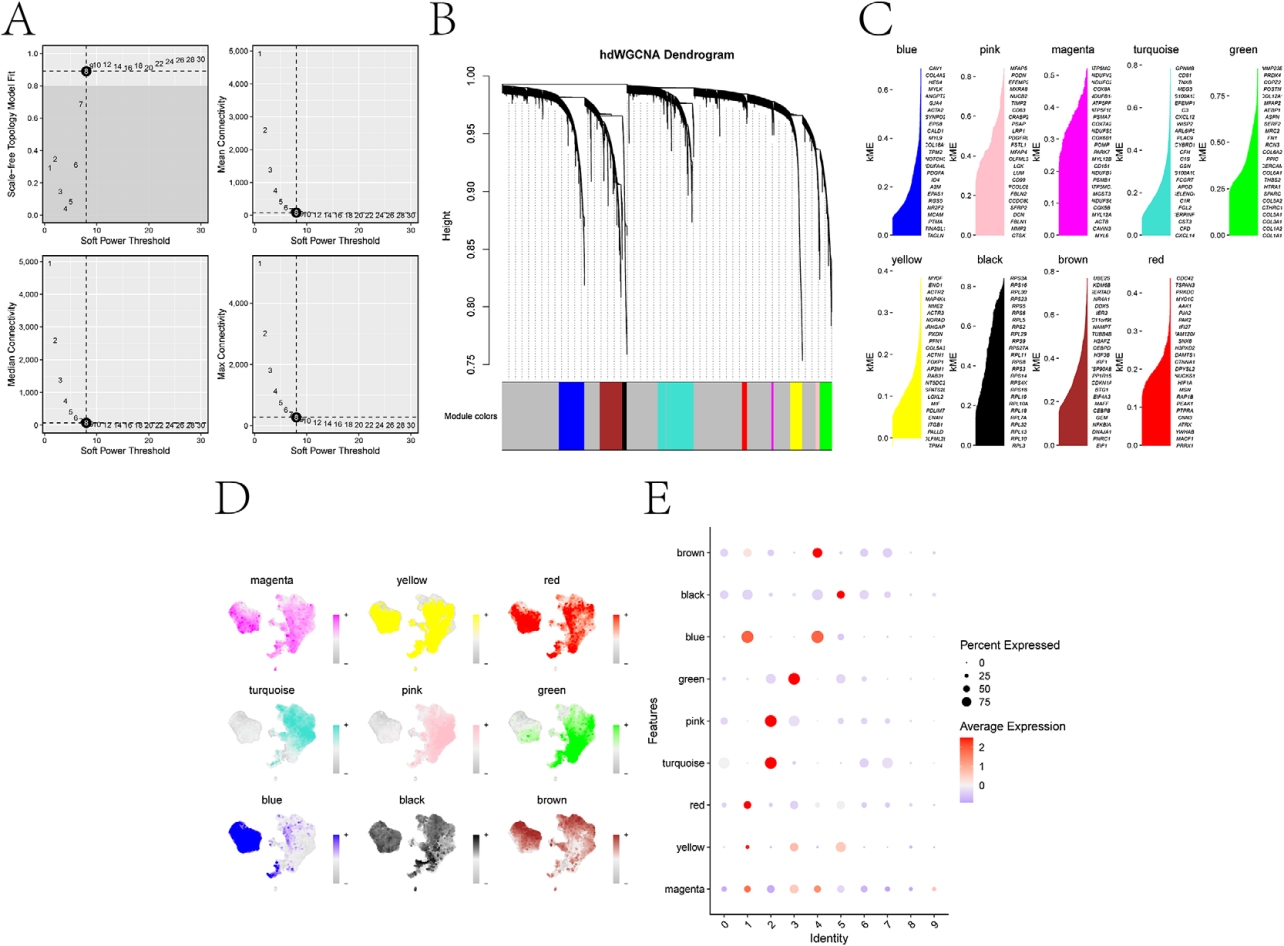
Single‐cell weighted gene co‐expression network analysis (hdWGCNA). (A) Scale‐free index and average connectivity of each soft threshold. (B) Dendrogram showing gene clustering, with different colors representing different modules. (C–D) The feature genes ranking high in different modules and the feature maps of module scores. (E) The correlation among the characteristic genes of the module.

To elucidate co‐expression patterns within fibroblast subtypes, we conducted hdWGCNA analysis. Subtype identities were specified using group.by as both cluster‐derived and custom‐defined labels. A soft‐threshold power of 8 was selected using the “TestSoftPowers function” (Figure 3A). A total of nine co‐expression modules were identified and color‐coded as follows: magenta, yellow, red, turquoise, pink, green, blue, black, and brown (Figure 3B). Module eigengene (ME) relationships were subsequently evaluated (Figure 3C,D), revealing that the green module exhibited the highest ME expression in disease‐associated fibroblasts (Figure 3E). Consequently, genes within the green module were shortlisted as candidate genes for further investigation (Table S2).

### 3.4 MR Analysis

**FIGURE 4:**
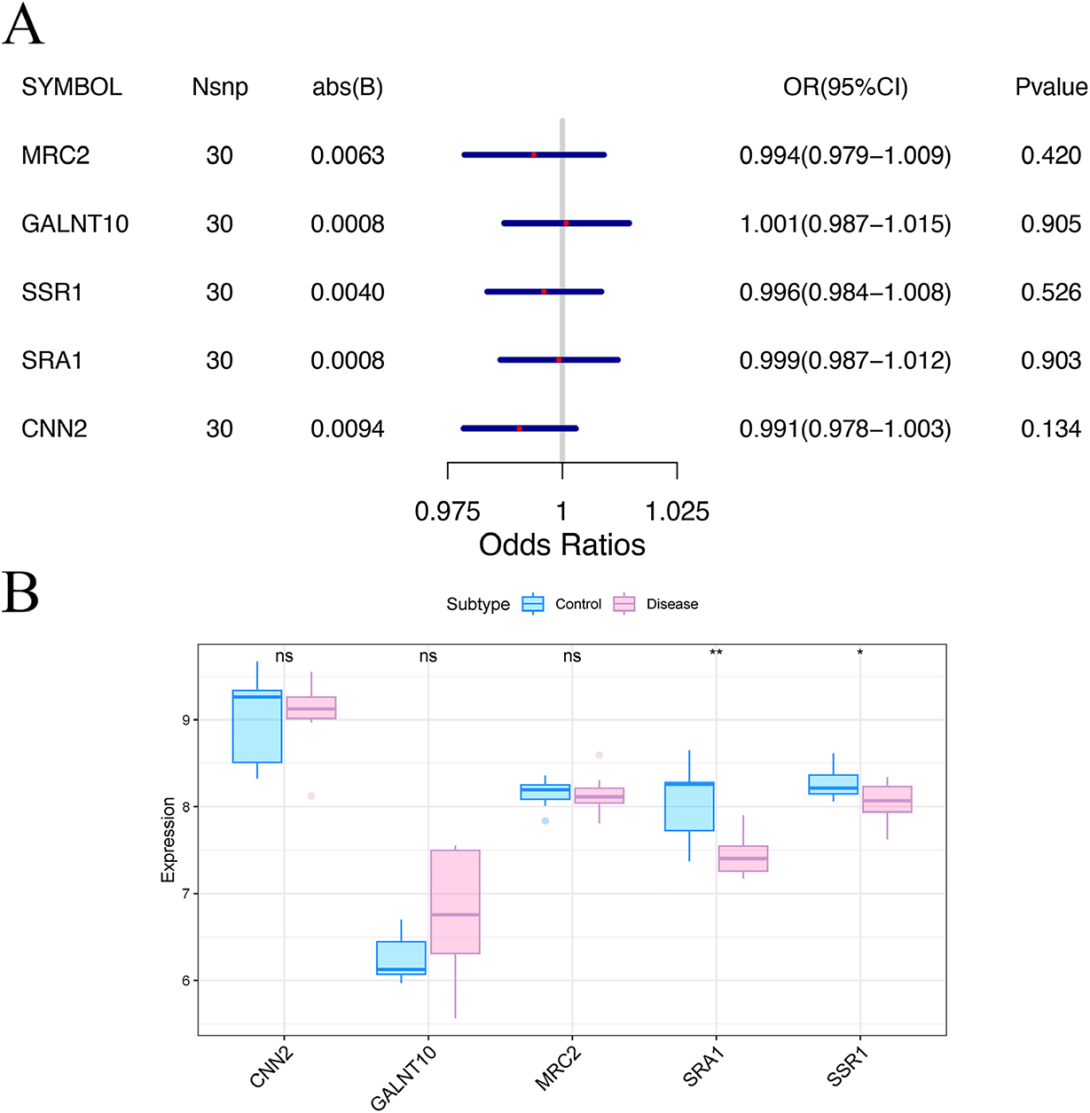
Reverse Mendelian randomization and differential expression analysis. (A) Reverse Mendelian randomization analysis for five key genes (MRC2, SSR1, CNN2, SRA1, and GALNT10), showing no evidence of reverse causality from disease risk to gene expression. (B) Box plots comparing the expression levels of the five genes between keloid samples and normal controls. Statistical significance is indicated where applicable.

To identify disease‐relevant genes from the selected modules, we performed MR analysis using GWAS summary statistics for keloid (GCST90018874; 668 cases and 481 244 controls; total 481 912 variants). Instrumental variables were extracted using extract_instruments function, and corresponding outcome data were obtained using extract_outcome_data. MR analysis identified 11 module genes with significant causal associations (inverse variance weighted [IVW] *p* < 0.05; Figure S3A): *MRC2*, *DAP*, *SSR1*, *C1QTNF3*, *NBL1*, *CNN2*, *SRA1*, *SAR1A*, *BAG2*, *LGALS1*, and *GALNT10*. Among them, nine genes were associated with decreased disease risk: *SSR1* (odds ratio [OR] = 0.737; 95% confidence interval [CI]: 0.601–0.904; *p* = 0.003), *C1QTNF3* (OR = 0.679; 95% CI: 0.482–0.955; *p* = 0.026), *NBL1* (OR = 0.671; 95% CI: 0.499–0.902; *p* = 0.008), *CNN2* (OR = 0.607; 95% CI: 0.376–0.981; *p* = 0.041), *SRA1* (OR = 0.605; 95% CI: 0.394–0.930; *p* = 0.022), *SAR1A* (OR = 0.563; 95% CI: 0.337–0.941; *p* = 0.028), BAG2 (OR = 0.551; 95% CI: 0.324–0.935; *p* = 0.027), *LGALS1* (OR = 0.470; 95% CI: 0.248–0.894; *p* = 0.021), and *GALNT10* (OR = 0.413; 95% CI: 0.174–0.983; *p* = 0.046). In contrast, *MRC2* (OR = 1.362; 95% CI: 1.073–1.729; *p* = 0.011) and *DAP* (OR = 1.104; 95% CI: 1.002–1.215; *p* = 0.046) were associated with increased disease risk. Leave‐one‐out sensitivity analysis confirmed the robustness of these findings, as the exclusion of any individual SNP did not significantly alter the causal estimates (Figure S3B–L). Subsequent colocalization analysis at the protein pQTL and GWAS level revealed strong shared signals (SNP.PP.H4 > 0.8) for *MRC2*, *SSR1*, *CNN2*, *SRA1*, and *GALNT10* (Figure S4A–E). However, reverse MR analysis found no evidence of reverse causality from these genes to disease risk (Figure 4A). Finally, analysis of bulk RNA‐seq data demonstrated that *SRA1* and *SSR1* showed significant expression differences between groups, consistent with the direction of effect observed in the MR results (Figure 4B). These findings highlight *SRA1* and *SSR1* as key candidate genes for downstream functional validation.

### 3.5 GSEA Analysis

**FIGURE 5:**
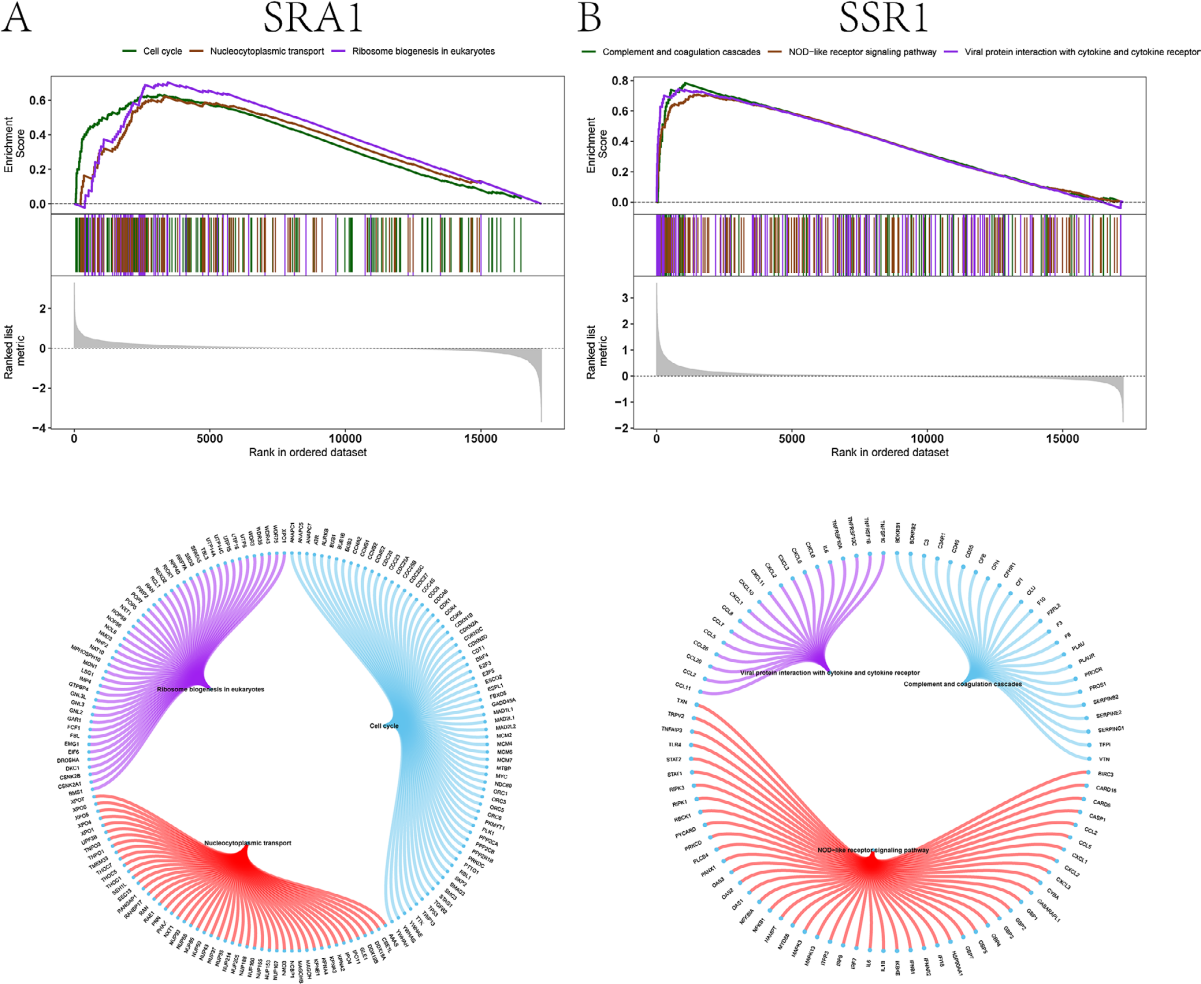
Gene Set Enrichment Analysis (GSEA) of key genes in keloid tissues. (A) Top: GSEA enrichment plots show significant positive enrichment of gene sets related to the cell cycle, nucleocytoplasmic transport, and ribosome biogenesis in *SRA1*‐high expressing cells. Bottom: The corresponding network plot (Cnet plot) depicts the interconnected gene networks driving these pathways. (B) Top: GSEA enrichment plots reveal significant positive enrichment of the complement and coagulation cascades, the NOD‐like receptor signaling pathway, and viral protein interactions with cytokines and cytokine receptors in *SSR1*‐high expressing cells. Bottom: The Cnet plot illustrates the immune‐related gene networks potentially regulated by *SSR1*.

To investigate the biological processes associated with key genes, we performed GSEA to identify relevant signaling pathways and elucidate their potential roles in disease pathogenesis. The results revealed that *SRA1* was predominantly enriched in pathways related to the cell cycle, nucleocytoplasmic transport, and ribosome biogenesis in eukaryotes (Figure 5A). The accompanying circular network (Cnet) plot visualizes the connections between these three pathways and their core constituent genes. In contrast, *SSR1* showed enrichment in pathways associated with the complement and coagulation cascades, the NOD‐like receptor signaling pathway, and viral protein interaction with cytokine and cytokine receptors (Figure 5B). The accompanying Cnet plot illustrates the connections between these pathways and their key genes.

### 3.6 Immunoinfiltration Analysis

**FIGURE 6:**
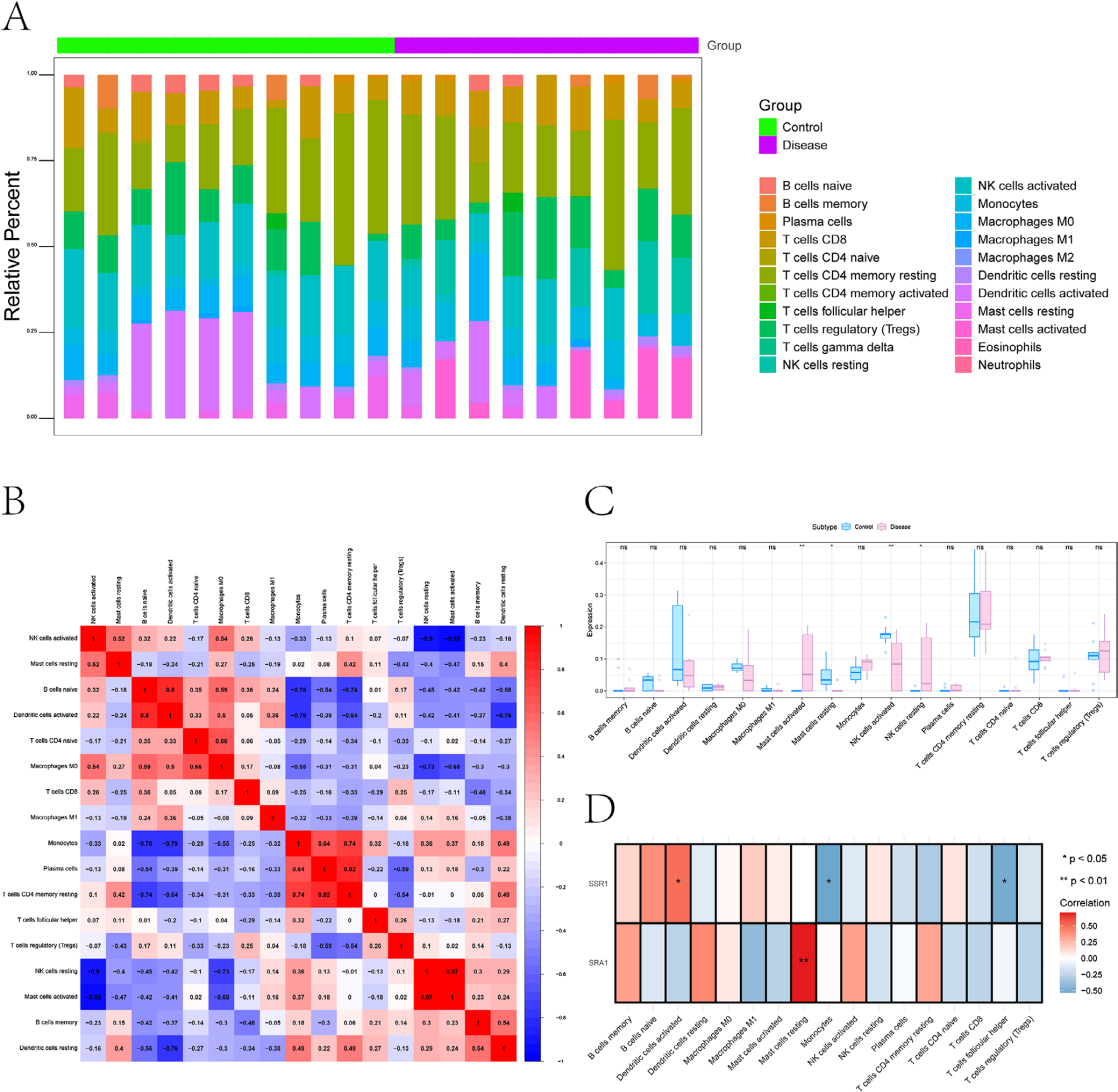
Immune infiltration landscape in keloid tissues. (A) Bar plot showing the relative abundance of 22 immune cell subsets across all samples. (B) Heatmap depicting the correlation matrix among immune cell types, where red indicates a positive correlation and blue indicates a negative correlation. (C) Heatmap comparing the estimated infiltration levels of 22 immune cell types between normal control and keloid samples. The color scale represents the relative abundance of each immune cell subset, with red indicating higher infiltration and blue indicating lower infiltration. (D) Heatmap illustrating the correlation between the expression levels of key genes (SRA1 and SSR1) and the abundance of various immune cell subsets. Red and blue represent positive and negative correlations, respectively (*p < 0.05, **p < 0.01).

The tissue microenvironment, which comprises fibroblasts, immune cells, extracellular matrix components, cytokines, growth factors, and unique biochemical features, plays a critical role in influencing disease progression, prognosis, and treatment response [13]. To characterize the immune landscape in keloid, we evaluated immune infiltration patterns and visualized both the distribution of immune cell populations and their interrelationships (Figure 6A,B). Compared with the control samples, keloid tissues exhibited higher levels of activated mast cells and resting natural killer (NK) cells, whereas resting mast cells and activated NK cells were reduced (Figure 6C). Correlation analyses further revealed that *SRA1* expression was significantly positively correlated with resting mast cells, while *SSR1* showed a positive association with activated dendritic cells and a negative correlation with monocyte levels (Figure 6D). We further assessed the relationships between the expression of key genes and various immune‐related factors, including chemokines, immune receptors, major histocompatibility complex (MHC) molecules, immunoinhibitors, and immunostimulators, highlighting their potential regulatory roles in immune infiltration and modulation of the tissue microenvironment (Figure S5A–E).

### 3.7 Transcription Factor Regulatory Network and Motif Enrichment Analysis of *SSR1* and *SRA1*

**FIGURE 7:**
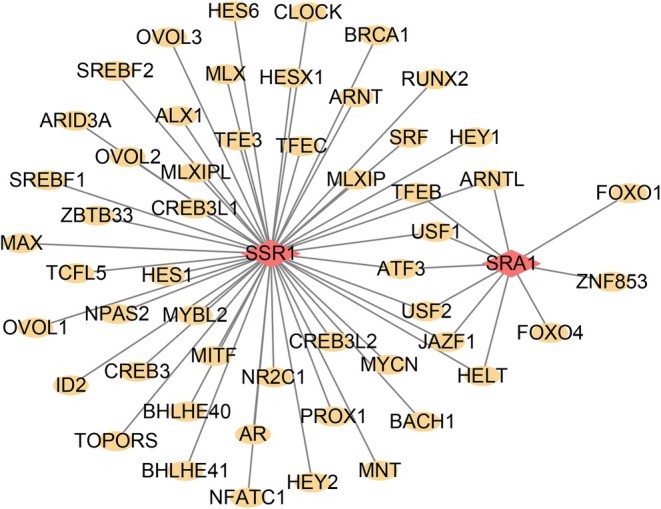
Transcriptional regulatory network and motif enrichment analysis of *SSR1* and *SRA1* in keloid tissues. Transcription factor regulatory network centered on *SSR1* and *SRA1*. Red nodes represent the key genes *SSR1* and *SRA1*, and gold nodes represent their interacting transcription factors.

To explore the potential transcriptional regulatory mechanisms underlying the identified key genes, a transcription factor (TF) regulatory network centered on *SSR1* and *SRA1* was constructed (Figure 7). This analysis revealed that these genes are commonly regulated by multiple transcription factors (TFs). Using cumulative recovery curves, we identified enriched transcription factors and matched motifs. The top five significantly enriched motifs, ranked by normalized enrichment score (NES), are presented in Table S3. Among the identified motifs, cisbp__M5038 exhibited the highest normalized enrichment score (NES = 5.77) and targeted *SSR1* exclusively. Predicted transcription factors (e.g., USF1, ARNT, CREB3L2) with potential binding sites in the regulatory regions of *SSR1* and *SRA1* are highlighted. Notably, USF1 was identified as a common regulator of both genes.

### 3.8 Expression Abundance and Quasi‐Time Series Analysis of Key Gene

**FIGURE 8:**
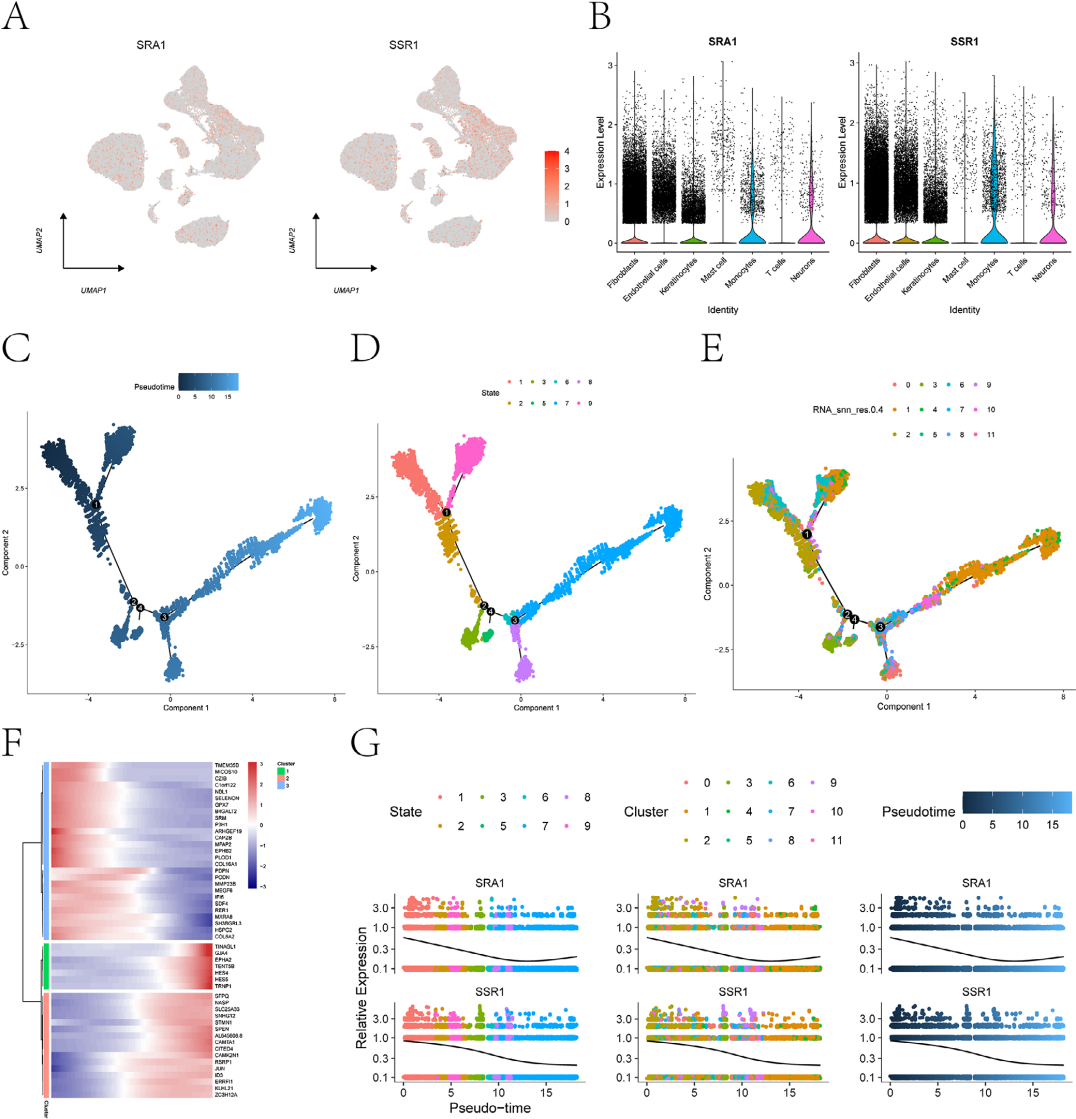
Single‐cell expression and pseudotemporal trajectory analysis of *SSR1* and *SRA1* in keloid tissues. (A) Dot plot showing the expression levels of SRA1 and SSR1 across major cell types. Dot size represents the proportion of expressing cells and color intensity represents the average expression level. (B) Violin plots displaying the expression distributions of SRA1 and SSR1 in major cell populations, including fibroblasts, endothelial cells, keratinocytes, mast cells, monocytes, T cells, and neurons. (C–E) Pseudotime trajectory inferred using Monocle. Cells are projected into a two‐dimensional space defined by Component 1 and Component 2. Colors indicate pseudotime progression (C), cellular states (D), and cell clusters (E). Black lines represent the inferred trajectory. (F) Branch heatmap showing genes with dynamic expression patterns across pseudotime branches. Red indicates higher expression and blue indicates lower expression. (G) Dynamic expression patterns of *SRA1* and *SSR1* along pseudotime.

To visualize the expression profiles of key genes across distinct cell populations, the DotPlot and FeaturePlot functions in the Seurat R package were employed. This analysis revealed that fibroblasts exhibited the highest expression levels of both *SRA1* and *SSR1* compared to other cell types, including endothelial cells, keratinocytes, mast cells, monocytes, T cells, and neurons (Figure 8A,B).

To investigate the dynamic transcriptional changes associated with cellular development during keloid formation, we performed pseudotime trajectory analysis using Monocle. We mapped and visualized the inferred temporal progression, including pseudotime ordering, cluster identity, and cellular differentiation states, illustrating the dynamic gene expression changes and differentiation trajectories of key cell populations throughout keloid formation (Figure 8C–E). Although branch heatmap analysis did not identify pronounced branch‐specific gene expression signatures, we detected genes exhibiting significant expression changes across distinct pseudotime intervals (Figure 8F). Additionally, the expression patterns of key genes were examined across different pseudotime stages, clusters, and cellular states (Figure 8G). Notably, both *SRA1* and *SSR1* were expressed during the early stages of keloid formation, with their expression levels progressively declining as the inferred disease progressed.

## 4 Discussion

A keloid is a pathological scar characterized by excessive fibroblast proliferation and dysregulated extracellular matrix deposition, exhibiting both “immunoinflammatory” and “neoplastic‐like” characteristics [2, 36, 37]. Despite extensive research, its underlying molecular mechanisms and core regulatory genes remain incompletely defined, hindering the development of targeted therapeutic strategies. In the present study, we systematically investigated the cellular heterogeneity, core regulatory genes, and underlying molecular mechanisms associated with keloid formation using a multi‐omics approach, including single‐cell RNA sequencing, high‐dimensional weighted gene co‐expression network analysis (hdWGCNA), MR, and GSEA.

Single‐cell transcriptomic profiling revealed that keloid tissues contained the seven major cell types (fibroblasts, endothelial cells, keratinocytes, mast cells, monocytes, T cells, and neurons) as those observed in normal scars. CellChat analysis further identified fibroblasts as the central hub of intercellular communication in keloid tissues, with extensive ligand–receptor interactions with endothelial cells, myeloid immune cells, and T cells. Among these, the most prominent signaling axes were MIF‐(CD74 + CD44) and PTN‐NCL. Macrophage migration inhibitory factor (MIF) is known to promote proliferation and inhibit apoptosis in fibroblasts by binding to CD74, which recruits CD44 and thereby activates the ERK1/2 signaling pathway [38], while pleiotrophin (PTN) is a secreted growth factor that binds to nucleolin (NCL) on the cell surface, triggering intracellular signaling cascades involved in cell migration, angiogenesis, and cytoskeletal reorganization [39]. Secondary clustering of fibroblasts identified 12 subtypes, with subtype 3 specifically enriched in disease samples, defining it as a disease‐specific population. This finding highlights the functional heterogeneity of fibroblasts in keloid [40].

Through hdWGCNA, we identified a disease‐associated fibroblast module, from which MR analysis pinpointed 11 genes with causal links to keloid risk. Notably, genetically determined downregulation of *SSR1* and *SRA1* was significantly associated with increased keloid susceptibility. *SRA1* and *SSR1* have not been previously reported in keloid research, with their functions primarily studied in tumors, obesity, cardiovascular disorders, and neurological disorders [41, 42, 43, 44]. GSEA analysis revealed their functional roles: *SRA1* was enriched in cell cycle, nucleocytoplasmic transport, and eukaryotic ribosome biogenesis pathways. These pathways are central to the regulation of cell proliferation, protein synthesis, and intracellular material transport [45, 46, 47]. In contrast, *SSR1* was enriched in complement and coagulation cascades, NOD‐like receptor signaling, and viral protein‐cytokine interactions, pointing to a function in modulating innate immunity, immune‐inflammatory responses, and tissue repair [48, 49, 50]. Interestingly, the identification of USF1 as a shared transcription factor suggests a potential transcriptional regulatory link between *SRA1* and *SSR1*. Previous studies have reported that USF1 regulates gene programs associated with immune responses and can transcriptionally activate TGF‐β signaling pathways [51, 52]. Therefore, dysregulation of the USF1‐centered transcriptional network may contribute to keloid pathogenesis by simultaneously affecting immune cell activity and profibrotic signaling. Additionally, pseudotemporal trajectory analysis suggested that both *SRA1* and *SSR1* were expressed during the early stages of keloid formation, with their expression levels progressively declining along the inferred disease progression trajectory. These findings suggest that these genes may function primarily during the initiation phase of keloid pathogenesis.

Furthermore, immunoinfiltration analysis showed that keloid tissues exhibited an imbalanced immune microenvironment relative to controls, with higher levels of activated mast cells and resting NK cells, and lower levels of resting mast cells and activated NK cells. These findings corroborate previous studies underscoring the role of activated mast cells in keloid pathogenesis, while also pointing to possible inhibition of NK cell activity [8, 13]. Activation of mast cells releases several inflammatory mediators, including TGF‐β, IL‐6, IL‐33, VEGF, PDGF, tryptase, and chymase, which contribute to the activation of keloid fibroblasts, increased collagen synthesis, angiogenesis, and enhance vascular permeability, thereby facilitating the infiltration of more inflammatory cells [53, 54]. In our study, a higher level of resting NK cells was found in keloid tissues, suggesting compromised adaptive immune responses and this immunomodulatory state potentially promotes the proliferation and development of keloid fibroblasts, similar to the functional exhaustion of natural killer cells observed in tumor microenvironments where neoplastic progression is uncontrolled [8, 55]. Correlation analyses further revealed that *SRA1* expression was significantly positively correlated with resting mast cells, further supporting the role of *SRA1* as a protective factor in keloid pathogenesis. This finding suggests that *SRA1* downregulation may contribute to keloid development by modulating mast cell activity. While *SSR1* showed a positive association with activated dendritic cells and a negative correlation with monocyte levels. The downregulation of *SSR1* observed in keloid tissues may therefore disrupt dendritic cell‐mediated immune regulation while promoting monocyte‐driven inflammation, both of which could facilitate fibrotic remodeling in keloid development. The extensive correlation analysis between key genes and five classes of immune regulators (chemokines, receptors, MHC molecules, immunoinhibitors, and immunostimulators) revealed that *SRA1* and *SSR1* may modulate the immune microenvironment through complex complementary mechanisms.

In conclusion, this integrative study identifies *SSR1* and *SRA1* as potential protective factors in keloid pathogenesis. These genes are enriched in keloid fibroblast populations and may function as key regulatory nodes during the initiation phase of keloid development, influencing cell proliferation, immune microenvironment remodeling, and cellular signaling pathways. Together, these findings advance the molecular understanding of keloid pathogenesis and highlight promising targets for future therapeutic investigation.

## 5 Strengths and Limitations

This study represents the first integrative analysis combining single‐cell transcriptomics (scRNA‐seq), protein quantitative trait loci (pQTL), and MR analyses to identify key genes and regulatory networks involved in keloid pathogenesis. Despite the methodological rigor of our integrated framework, this study is limited by the absence of experimental validation through in vitro and in vivo models. The proposed suppressive roles of *SRA1* and *SSR1* in keloid development are currently supported by correlational evidence and therefore require functional confirmation. Future studies involving knockdown or overexpression of *SRA1* and *SSR1* in keloid fibroblasts, followed by assessment of cellular phenotypes and validation in animal models, will be essential to establish their functional significance in keloid pathogenesis.

## Author Contributions

Yu‐Kun Zhao and Hui‐Hui Wu designed and conducted the whole study. Yu‐Kun Zhao, Juan‐Hua Liu, Mu‐Kai Chen, Di‐Qing Luo, and Yong‐Xia Meng applied for the data analysis. Yu‐Kun Zhao, Hui‐Hui Wu, and Yong‐Xia Meng finalized the manuscript. Yu‐Kun Zhao made outstanding contributions to the further improvement of the research and the revision of the manuscript. All authors contributed to the article and approved the submitted version.

## Funding

This work was supported by the National Natural Science Foundation of China (Grant 82304059).

## Ethics Statement

The authors have nothing to report.

## Consent

The authors have nothing to report.

## Conflicts of Interest

The authors declare no conflicts of interest.

## Supporting information


**Figure S1:** Pre‐treatment of single cells.
**Figure S2:** Secondary clustering.
**Figure S3:** Mendelian randomization and leave‐one‐out sensitivity analysis.
**Figure S4:** Co‐localization analysis of key genes.
**Figure S5:** Correlation of key genes with immune regulatory factors in keloid.

**Table S1:** Clinical characteristics of samples included in the single‐cell RNA sequencing datasets GSE181297 and GSE163973.

**Table S2:** xlsx. Candidate genes from the green co‐expression module in disease‐associated fibroblasts.

**Table S3:** Top five enriched transcription factor binding motifs associated with *SSR1* and *SRA1* ranked by normalized enrichment score (NES).

## Data Availability

The data is available in the Gene Expression Omnibus (GEO) public database (https://www.ncbi.nlm.nih.gov/geo/info/datasets.html) and pQTL data from the deCODE database (https://www.decode.com/summarydata/). Further inquiries can be directed to the corresponding author.
