# Supplementary material for: Study on the Potential Molecular Mechanism of Keloid Disease Associated With Single Cell Combined Mendelian Randomization
Source: J Cosmet Dermatol. 2026 Apr 15;25(4):e70849. doi: 10.1111/jocd.70849 (PMC13083048; doi:10.1111/jocd.70849)

**Supplementary figure legends**

**Supplementary Figure 1: Pre-treatment of single cells**

(A) Single-cell quality control, showing the number of cells, genes and sequencing depth of each sample.

(B) The left graph shows the relationship between cell sequencing depth and mitochondrial content, and the right graph shows the relationship between sequencing depth and the number of genes. The scatter plot shows the correlation between mitochondrial content (Y-axis) and nCount_RNA (X-axis). Each dot represents a cell, indicating the distribution of RNA count relative to the expression level of mitochondrial genes.

(C) We identified the genes with significant differences among cells and plotted the characteristic variance plots.

(D) Variance ranking graph of each PC.

The display of (E-F)PCA and the distribution of PC, with points representing cells and colors representing samples.


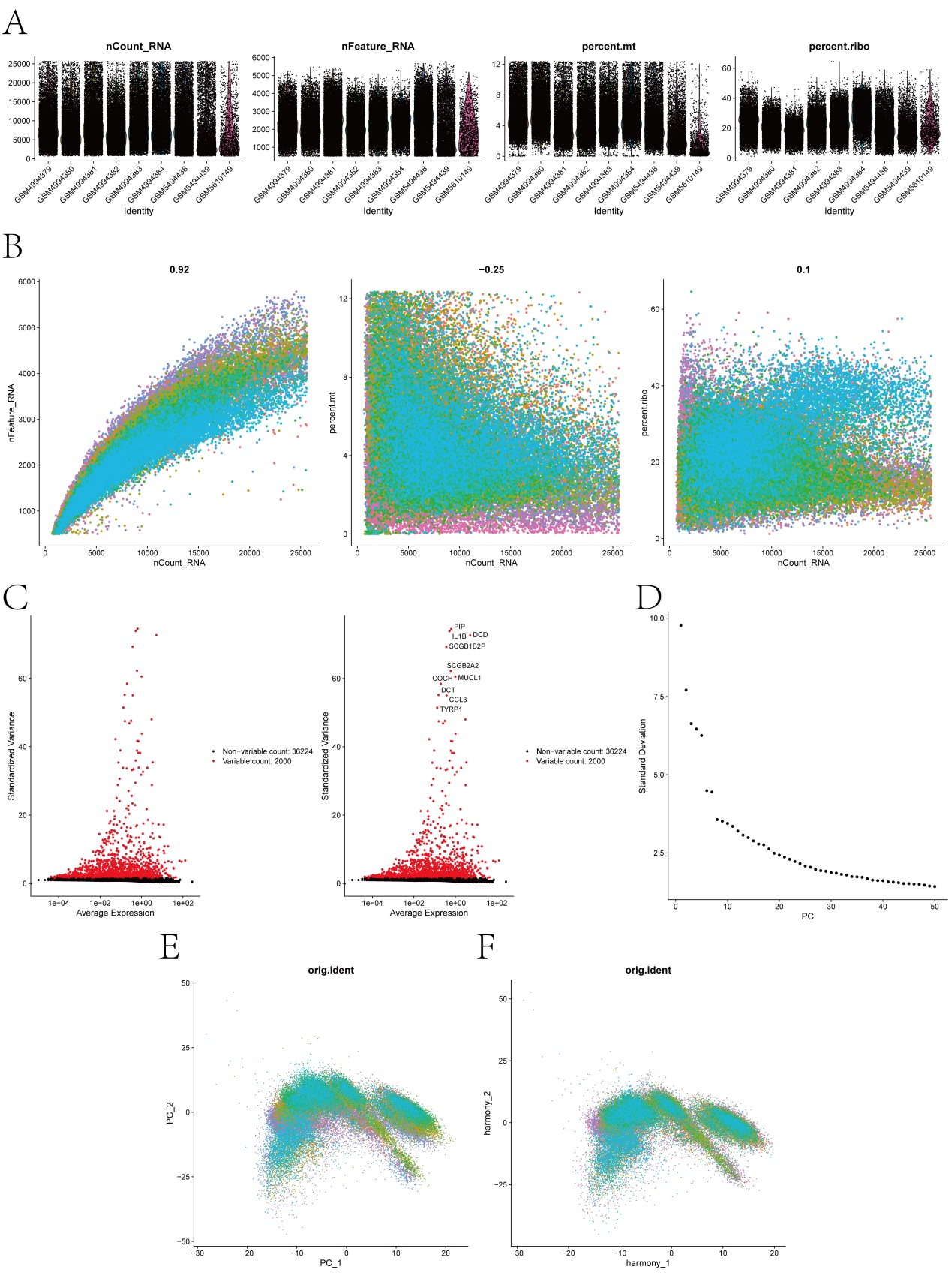


**Supplementary Figure 2: Secondary clustering**

(A) The variance ranking graph of each PC in the secondary clustering.

(B-C) The display of secondary clustering PCA and the distribution of PC, where points represent cells and colors represent samples.

(D) Secondary clustering: Based on the important components available in PCA, cells are divided into 12 clusters through the UMAP algorithm.

(E) The difference in the proportion of 12cluster in the two groups of samples**.**


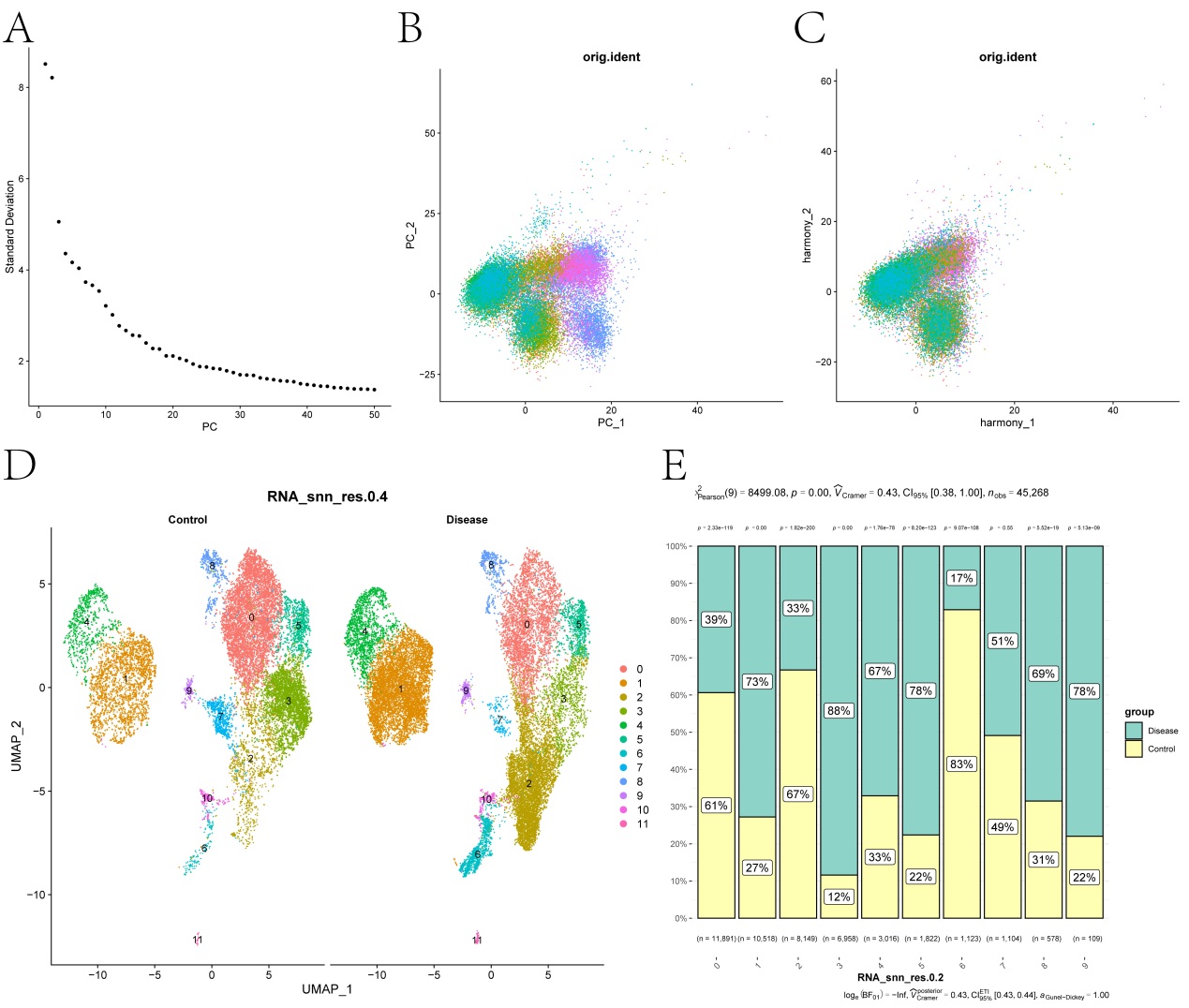


**Supplementary Figure 3. Mendelian Randomization and Leave-One-Out Sensitivity Analysis**

(A) Forest plot summarizing the causal effects of 11 genetically predicted genes on keloid risk, as estimated by Mendelian randomization analysis.
(B-L) Leave-one-out sensitivity analyses for each of the 11 genes, showing the impact of excluding individual SNPs on the overall causal estimate.


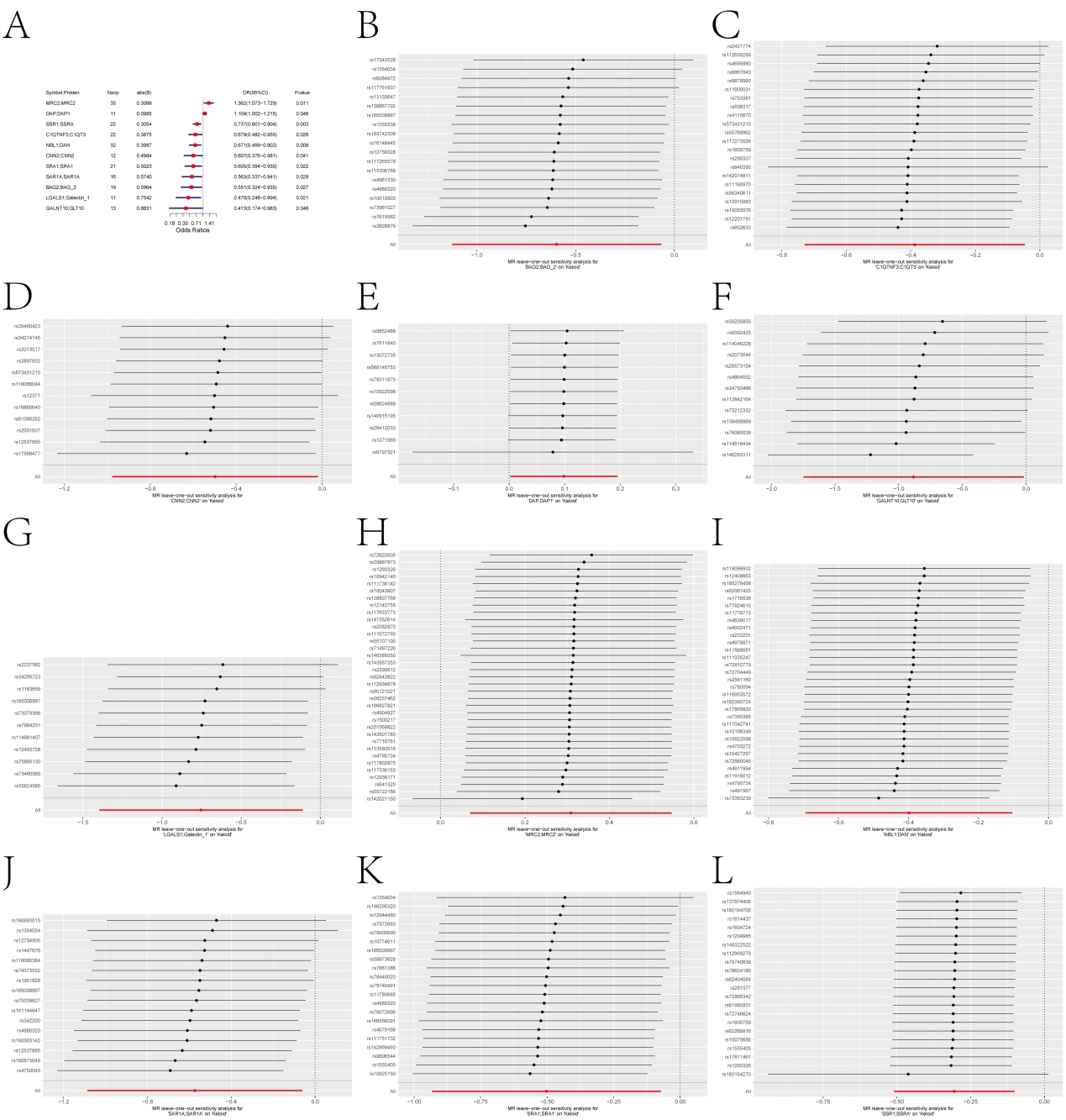


**Supplementary Figure 4. Co-localization Analysis of Key Genes**

(A–E) Co-localization plots for five key genes (*MRC2*, *SSR1*, *CNN2*, *SRA1*, and *GALNT10*). Each point represents a single nucleotide polymorphism (SNP), with its position on the x‑axis reflecting the −log₁₀(P value) from the genome-wide association study (GWAS) for keloid, and its position on the y‑axis reflecting the −log₁₀(P value) from the protein quantitative trait locus (pQTL) analysis. SNPs showing strong evidence of co-localization (SNP.PP.H4 > 0.8) are highlighted, indicating a shared causal variant between gene expression and keloid disease risk.


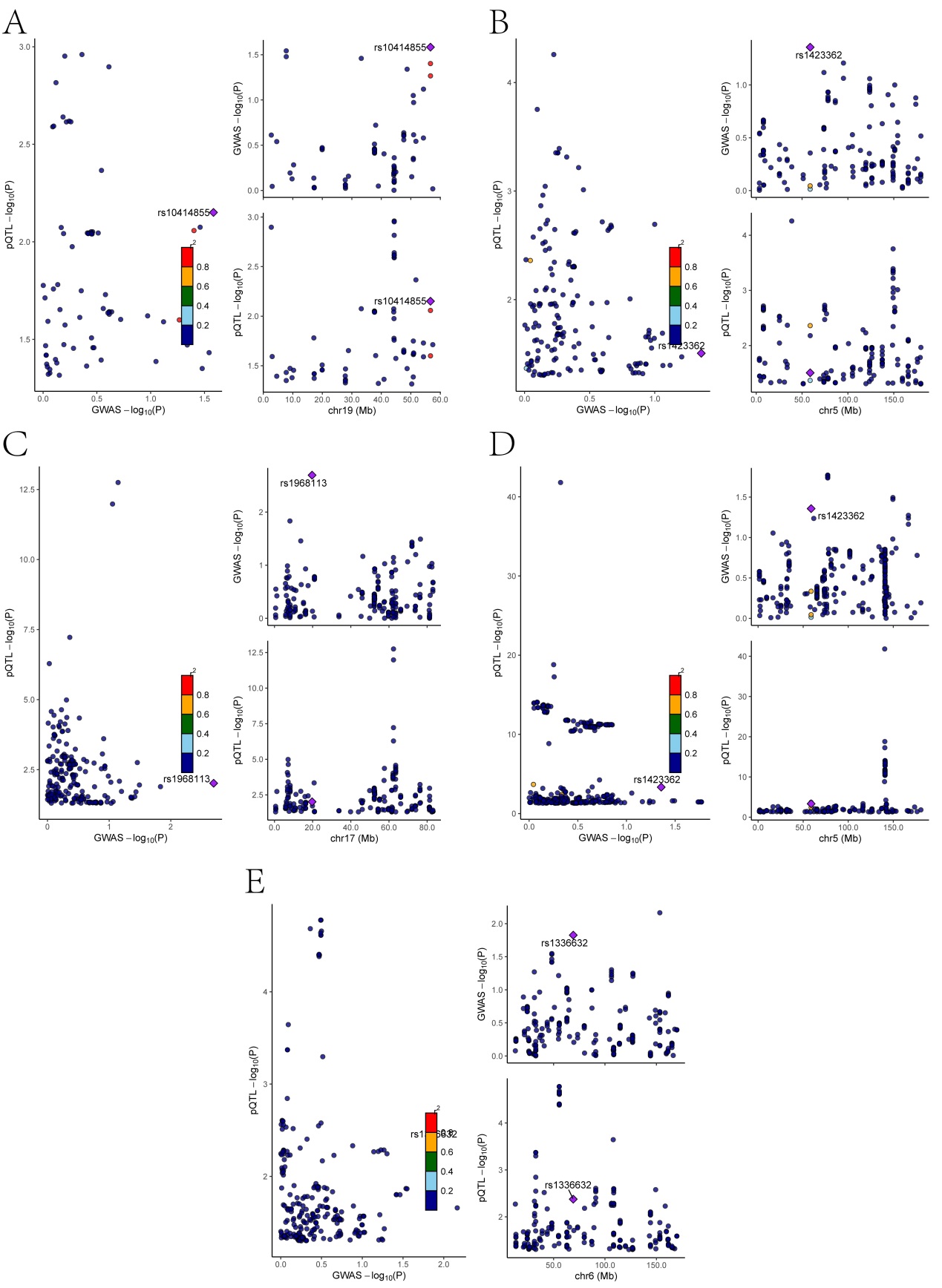


**Supplementary Figure 5. Correlation of Key Genes with Immune Regulatory Factors in Keloid**

(A–E) Heatmaps showing the correlation between the expression levels of key genes (*SRA1* and *SSR1*) and five classes of immune regulatory factors: (A) chemokines, (B) immunoinhibitors, (C) immunostimulators, (D) major histocompatibility complex (MHC) molecules, and (E) immune receptors. Red indicates a positive correlation, blue indicates a negative correlation, and the color intensity reflects the strength of the correlation. Statistical significance is denoted where applicable.


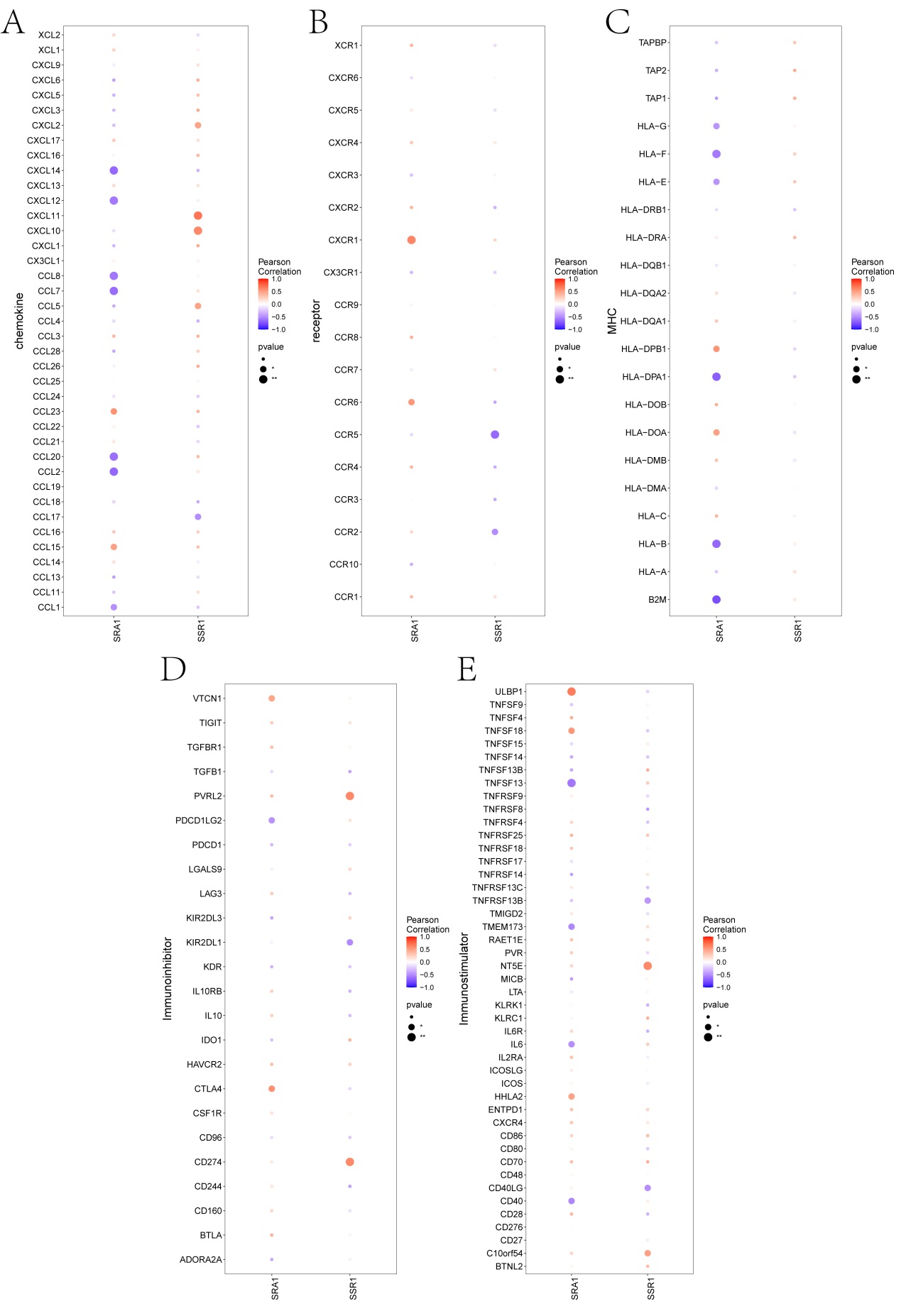

Supplement: Supplementary file 1 — Figure S1: Pre‐treatment of single cells. Figure S2: Secondary clustering. Figure S3: Mendelian randomization and leave‐one‐out sensitivity analysis. Figure S4: Co‐localization analysis of key genes. Figure S5: Correlation of key genes with immune regulatory factors in keloid. [file JOCD-25-e70849-s004.docx]
