# Supplementary material for: Study on the Potential Molecular Mechanism of Keloid Disease Associated With Single Cell Combined Mendelian Randomization
Source: J Cosmet Dermatol. 2026 Apr 15;25(4):e70849. doi: 10.1111/jocd.70849 (PMC13083048; doi:10.1111/jocd.70849)
Supplement: Supplementary file 2 — Table S1: Clinical characteristics of samples included in the single‐cell RNA sequencing datasets GSE181297 and GSE163973. [file JOCD-25-e70849-s003.docx]

| Sample ID | | Tissue collected | Age | Sex | Race | Site of Sampling | Intralesional  steroids prior to  excision |
| --- | --- | --- | --- | --- | --- | --- | --- |
| GSE181297 | GSM5494438 | keloid | 23 | Male | Asian | ear | no |
|  | GSM5494439 | keloid | 25 | Male | Asian | back | no |
|  | GSM5610149 | normal scar | 25 | Male | Asian | back | no |
| GSE163973 | GSM4994379 | keloid | 20 | Male | Han nationality | back | no |
|  | GSM4994380 | keloid | 23 | Male | Han nationality | chest | no |
|  | GSM4994381 | keloid | 34 | Female | Han nationality | chest | no |
|  | GSM4994382 | normal scar | 39 | Male | Han nationality | back | no |
|  | GSM4994383 | normal scar | 28 | Male | Han nationality | chest | no |
|  | GSM4994384 | normal scar | 26 | Female | Han nationality | chest | no |

**Supplementary Table 1. Clinical characteristics of samples included in the single-cell RNA sequencing datasets GSE181297 and GSE163973.**
