# Supplementary material for: Study on the Potential Molecular Mechanism of Keloid Disease Associated With Single Cell Combined Mendelian Randomization
Source: J Cosmet Dermatol. 2026 Apr 15;25(4):e70849. doi: 10.1111/jocd.70849 (PMC13083048; doi:10.1111/jocd.70849)
Supplement: Supplementary file 4 — Table S3: Top five enriched transcription factor binding motifs associated with SSR1 and SRA1 ranked by normalized enrichment score (NES). [file JOCD-25-e70849-s001.docx]

**Supplementary Table 3. Top five enriched transcription factor binding motifs associated with *SSR1* and *SRA1* ranked by normalized enrichment score (NES).**

| Number | Motif | NES | AUC | TF_highConf | nEnrGenes | enrichedGenes |
| --- | --- | --- | --- | --- | --- | --- |
| 1 | cisbp_M5038 | 5.77 | 0.483 |  | 1 | SSR1 |
| 2 | cisbp_M4922 | 5.76 | 0.482 |  | 1 | SSR1 |
| 3 | cisbp_M5997 | 5.75 | 0.482 | CREB3L2  (inferredBy_Orthology). | 1 | SSR1 |
| 4 | cisbp_M4637 | 5.72 | 0.479 | USF1 (directAnnotation). | 2 | SRA1, SSR1 |
| 5 | cisbp_M2956 | 5.71 | 0.478 | ARNT (directAnnotation). | 1 | SSR1 |
